# Different Dimensions of Affective Processing in Patients With Irritable Bowel Syndrome: A Multi-Center Cross-Sectional Study

**DOI:** 10.3389/fpsyg.2021.625381

**Published:** 2021-03-29

**Authors:** Sabrina Berens, Rainer Schaefert, Johannes C. Ehrenthal, David Baumeister, Wolfgang Eich, Jonas Tesarz

**Affiliations:** ^1^Faculty of Behavioural and Cultural Studies, Institute of Psychology, Heidelberg University, Heidelberg, Germany; ^2^Department of General Internal Medicine and Psychosomatics, University Hospital Heidelberg, Heidelberg University, Heidelberg, Germany; ^3^Department of Psychosomatic Medicine, Division of Internal Medicine, University Hospital Basel, Basel, Switzerland; ^4^Faculty of Medicine, University of Basel, Basel, Switzerland; ^5^Department of Psychology, University of Cologne, Cologne, Germany

**Keywords:** affect tolerance, alexithymia, emotion regulation, emotional awareness, functional somatic syndromes, somatization

## Abstract

**Objective:** Deficits in affective processing are associated with impairments in both mental and physical health. The role of affective processing in patients with functional somatic complaints such as irritable bowel syndrome (IBS) remains unclear. Most studies have focused on the capacity for emotional awareness and expression, but neglect other dimensions of affective processing. Therefore, this study aimed to systematically analyze differences in six different dimensions of affective processing between patients with IBS and healthy controls (HCs). Additionally, we exploratively investigated the impact of IBS symptom severity, psychological distress, and attachment styles on affective processing in IBS.

**Methods:** A controlled cross-sectional multi-center study was conducted. Overall, 127 patients with IBS were compared with 127 matched HCs using multivariate analysis of variances. Affective processing was operationalized in line with the affect cascade model on six specific dimensions: emotional experience, emotional awareness, affect tolerance, affect differentiation, affect regulation, and emotional communication. They were measured using two subscales of the Mentalizing Questionnaire (MZQ) and four subscales of the Operationalized Psychodynamic Diagnosis–Structure Questionnaire (OPD-SQ). Linear regression analysis was used to investigate the influence of IBS symptom severity (IBS-Severity Scoring System, IBS-SSS), depression (Patient Health Questionnaire, PHQ-9), anxiety (General Anxiety Disorder, GAD-7), and anxious and avoidant attachment styles (Experiences in Close Relationships Scale, ECR-RD12) on the different dimensions of affective processing in IBS.

**Results:** Patients with IBS compared to HCs showed deficits in all six dimensions of affective processing. Deficits were largest for affect tolerance (*d* = 0.849) and lowest for emotional experience (*d* = 0.222) and emotional awareness (*d* = 0.420). Moderate effect sizes were found for affect differentiation (*d* = 0.773), emotional communication (*d* = 0.665), and affect regulation (*d* = 0.552). Moreover, explorative analyses indicated that affective processing in patients with IBS was significantly influenced by levels of anxiety and insecure attachment.

**Conclusion:** The results indicate a specific pattern of affective processing abilities in patients with IBS. The deficits in affective processing are more prominent in the area of understanding and tolerating difficult affective states than experiencing affective states. This opens interesting perspectives for the development of specific psychotherapeutic interventions.

**Clinical Trial Registration:** DRKS00011685.

## Introduction

Irritable bowel syndrome (IBS) is a chronic gastrointestinal (GI) disorder that is characterized by disturbed gut-brain interactions (Drossman, 2016), resulting in abdominal pain and changes in bowel habits (Canavan et al., 2014a). IBS is one of the most frequent disorders presented to gastroenterology services (Soares, 2014) creating high socioeconomic costs (Canavan et al., 2014b) and impaired quality of life (Agarwal and Spiegel, 2011). The biopsychosocial disease model of IBS suggests a complex interaction of environmental, psychological, and biological factors with bidirectional interactions of the brain-gut axis (Oudenhove et al., 2016). As IBS symptom severity increases, central nervous system is more involved and psychosocial variables gain importance (Drossman et al., 2011). For these patients, psychotherapeutic interventions represent an important treatment element yet they achieve only small to moderate effects in reducing gastrointestinal symptoms (Martin et al., 2013). This means new strategies for potential treatment improvements are needed.

By considering IBS from a psychosocial perspective, the concept largely overlaps with concepts of functional somatoform and somatic symptom disorders (Hausteiner-Wiehle and Henningsen, 2014). Functional somatoform disorders are classically conceptualized as disorders of affect regulation (Waller and Scheidt, 2006) with deficits in embodied mentalization (Luyten et al., 2012). In line with developmental theories of attachment and mentalizing abilities, affect regulations strategies are shaped by early interactions between the child and a caregiver (Waller and Scheidt, 2006; Luyten et al., 2012; Fonagy et al., 2018). The capacity of the caregiver to adequately capture, process, and mirror the child's physical and affective states strongly influences the capacity of the child to differentiate, tolerate and communicate different affective states (Fonagy et al., 2018). If the process of affective tuning is impaired, this can result in downregulation and suppression of emotions in the context of an avoidant attachment style or in emotional overactivation in the context of an anxious attachment style (Mikulincer et al., 2003). Difficulties in understanding own affective states may result in somatic experience of emotional distress and increase the risk of functional somatoform disorders (Riem et al., 2018). According to Pennebaker's model of inhibition emotional expression and physiological arousal are inversely linked, so that a suppression of emotionally adverse experiences produces physiological strain and over time stress-related diseases (Pennebaker, 1989). In patients with IBS, this leads to modified neuronal emotional modulation in response to visceral stimuli (Elsenbruch et al., 2010) and impaired stress response (Chang, 2011).

One of the most well-researched concepts regarding affective processing is alexithymia (Sifneos, 1973), the difficulty of identifying and describing feelings along with the misinterpretation of bodily sensations that accompany emotional arousal (Taylor et al., 1999). Alexithymic traits have been shown to be especially relevant in functional gastrointestinal disorders (Porcelli et al., 1999; Kano et al., 2018). Patients with IBS have been shown to be more alexithymic than healthy controls (HCs) (Portincasa et al., 2003; Jones et al., 2006), and high alexithymia is associated with greater symptom severity (Porcelli et al., 2014) and worse treatment outcomes in IBS (Porcelli et al., 2003). Although there is now a body of evidence supporting the association of alexithymia and functional body complaints, to date few studies have analyzed the different dimensions of affective processing in a more differentiated manner. The studies that are available do suggest that the main deficits of affective processing are in differentiating emotions from bodily sensations (Faramarzi et al., 2015; Fournier et al., 2018). Building on such findings, novel psychotherapeutic approaches for patients with IBS focus specifically on aspects of emotional awareness (Farnam et al., 2014) as well as emotional awareness and expression (Thakur et al., 2017). These new therapeutic approaches show promising results and underline the relevance of affective processing in the treatment of patients with IBS.

Overall, there is consistent evidence for deficits in emotional awareness and expression in patients with IBS. Importantly, other dimensions of disturbed affective processing besides emotional awareness and expression have been described, but systematic studies on them are lacking to date. Current psychological concepts understand affective processing as the ability to mentalize inner states or as a structural deficit in personality functioning. These are broader concepts than the concept of alexithymia that integrate the complexity and multidimensionality of affective processing. Based on this, a sequential affect cascade model has been conceptualized by Rudolf (Rudolf, 2013). In accordance, this study distinguished six dimensions of disturbed affective processing in consecutive sequence: (1) generating (emotional experiencing), (2) perceiving (emotional awareness), (3) enduring (affect tolerance), (4) understanding (affect differentiation), (5) modulating (emotion regulation), and (6) expression (emotional communication). Each of these dimensions can be impaired and lead individually or together to a disturbed process of affective processing.

Accordingly, patients can have difficulties in *experiencing emotions* (1) in general [as a deficit in embodied affectivity (Fuchs and Koch, 2014) or as a kind of “emotional numbness” e.g., due to abuse and dissociation (Salmon et al., 2003)]; Additionally, patients may lack *emotional awareness* (2) [e.g., dysfunctional interoceptive awareness (Cameron, 2001) due to deficits in embodied mentalization in early infancy (Fotopoulou and Tsakiris, 2017; Fonagy et al., 2018) or emotional suppression (Scheier and Bridges, 1995)]. Furthermore, they may have difficulties in enduring emotions because they are flooded by excessive arousal and therefore may have deficits in *affect tolerance* (4) (Krystal, 1975) [e.g., due to a mismatch with early caregiver regarding affect containing and mirroring (Fonagy et al., 2018) or in general altered stress axis and hypersensitivity e.g., due to childhood trauma (Oudenhove et al., 2016). The ability to understand and classify one's own emotions is necessary (Ballespí et al., 2019) [*affect differentiation (3)*]. Deficits emerge if patients experience emotions (e.g., as unspecific arousal), but cannot differentiate and label them further, e.g., due to early lack of affective reflections by the early caregiver (Taylor and Bagby, 2000)/structural problem of bodily and emotional representations (Rudolf and Henningsen, 2003; Stonnington et al., 2013). There may also be deficits in emotion regulation (5) [e.g., lacking the ability to self-calm or dysfunctional suppression and avoidance strategies (Jones et al., 2006)]. Finally, there may be deficits in *expressing and communicating emotions to others* (6) [e.g., due to negative beliefs about emotions as a weakness (Bowers and Wroe, 2016), learned reinforcements of getting attention by communicating physical symptoms instead of affective states (Lind et al., 2014; Oudenhove et al., 2016), low early sensitivity of the caregiver regarding a family discourse about emotion (Harris, 1999), or deactivating attachment styles (Luyten et al., 2012)].

Overall, the model integrates bottom-up levels of affective processing (generating and perceiving) and top-down abilities of understanding and modulating affects (affect differentiation and emotion regulation) (Van den Bergh et al., 2014). Additionally, deficits in generating and perceiving emotions could be part of a deactivation regulation strategy regarding emotional and attachment related processes, while deficits in affect tolerance and regulation could be developmentally related to emotional hyperactivation (Mikulincer et al., 2003). It can be distinguished if intrapersonal deficits in affective processing or interpersonal expression and emotional communication is limited.

Although deficits in affective processing have been shown in numerous studies and new therapeutic interventions specifically address emotional processing, there is a lack of studies that systematically investigate the specific deficits of affective processing in patients with IBS. Therefore, this study aimed to analyze six different dimensions of affective processing between patients with IBS and HCs and to explore the extent of the deficits by calculating effect sizes. We hypothesized that (1.) patients with IBS are characterized by generally stronger deficits in affective processing compared to HCs; and in line with the established concept of alexithymia in somatoform disorders, that (2.) deficits are more pronounced in the area of emotional awareness and experiencing than in the other dimensions. Additionally, we exploratively investigated the impact of IBS symptom severity, psychological distress, and attachment styles on the different dimensions of affective processing in IBS.

## Materials and Methods

### Study Design and Patient Recruitment

This multi-center study compared patients with IBS with HCs according to different dimensions of affective processing. Participants were recruited between February and December 2017 in general practitioners' practices (primary care), gastroenterological specialty practices (secondary care) and outpatient clinics of the Department of General Internal Medicine and Psychosomatics at Heidelberg University Hospital. Healthy participants were recruited from the general population via an online opportunity sample by SoSciSurvey (Leiner, 2014). Before entering the study, all participants provided written and informed consent. The study was approved by the Ethics Research Committee of the Faculty of Medicine, University of Heidelberg (S-635/2016), and was carried out in compliance with the Helsinki Declaration. The study was registered by the German Clinical Trials Register (DRKS) (DRKS00011685). It is part of a larger project to investigate psychodynamic characteristics of patients with functional somatic symptoms (funded by the Köhler-Stiftung). First results have recently been published (Berens et al., 2019), for study flow chart see Berens et al. (2020).

### Inclusion and Exclusion Criteria

All patients had to be between 18 and 65 years old. Exclusion criteria were: inability to read, write and speak the German language; severe cognitive deficits or disabilities; acute severe organic disease or physical injury that hinders study participation; and acute psychosis. We combined questionnaire data with an assessment by a physician in our study to ensure diagnosis: Patients with IBS had firstly to fulfill the Rome III IBS symptom pattern (Longstreth et al., 2006), secondly the diagnosis had to be confirmed by a specialist during a clinical evaluation. The clinical evaluation included at least an anamnesis and, if necessary, further examination or diagnostic exclusion (e.g., patients with inflammatory bowel diseases were excluded). Participants from the healthy controls were excluded if they fulfilled the Rome III IBS symptom pattern, if they reported to be diagnosed with IBD, IBS or any psychiatric disorder, or take antidepressants.

### Measurements of IBS Characterization

Rome III criteria for IBS were considered met, if recurrent abdominal pain or discomfort was reported on at least 3 days per month in the past 3 months (Longstreth et al., 2006). Additionally, the complaints had to be associated with two or more of the following symptoms: improvement with defecation, change in stool frequency or change in stool form. Symptoms had to be chronic (at least for 6 months). IBS subtypes according to ROME III criteria included IBS-C (constipation), IBS-D (diarrhea), IBS-M (mixed), and IBS-U (undefined).

IBS symptom severity was assessed using the irritable bowel severity scoring system section one (*IBS-SSS*; range 0–500) (Francis et al., 1997). The questionnaire assesses pain severity and duration, distension, satisfaction with bowel habits and impairment due to symptoms. A sum score (range 0–500) as well as severity levels can be classified: mild (<175), moderate (175–300), and severe (>300).

### Measurements of Affective Processing

The primary objective of this study was to investigate different dimensions of affective processing and to identify specific patterns of affective processing deficits in patients with IBS compared to HCs. Although various questionnaires exist for assessing specific dimensions of affective processing (e.g., emotion regulation), so far there is no single instrument that assesses affective processing in a structured way across the different dimensions of affective processing. In line with the sequential affect cascade model suggested by Rudolf (Rudolf, 2013), we aimed to cover the whole spectrum of affective processing from generating, perceiving, tolerating, understanding, modulating, and expressing of affects. To achieve this, we opted to combine six different subscales of two well-validated assessment instruments to balance the competing demands of using validated assessment instruments and providing a thorough evaluation of the different dimensions of affective processing. Therefore, the sum scales of two subscales of the Mentalizing Questionnaire (MZQ) (Hausberg et al., 2012) and four subscales of the Operationalized Psychodynamic Diagnosis–Structure Questionnaire (OPD-SQ) (Ehrenthal et al., 2012) were analyzed. All items had an answering format of 0–4. Higher values reflect higher deficits in affective processing.

- *Emotional experience* is a four-item subscale of the OPD-SQ (e.g., “I notice that important events don't really make me feel anything.”). It is the ability of emotional communication with oneself and describes the capacity to be emotionally affected. The scale showed excellent reliability (Cronbach's α = 0.831) in our study.
- *Emotional awareness* is one of four subscales of the MZQ that consists of four items (e.g., “Often I don't even know what is happening inside of me”). It describes the inability to perceive one's own inner states which is a central aspect of mentalization (Hausberg et al., 2012). The scale showed good reliability (Cronbach's α = 0.771) in our study.
- *Affect differentiation* is a four-item subscale of the OPD-SQ (e.g., “It's often unclear to me what exactly I'm feeling right now”). It is the ability of self-awareness and describes the capacity to differentiate between different emotional states. The scale showed excellent reliability (Cronbach's α = 0.849) in our study.
- *Affect tolerance* is a five-item subscale of the OPD-SQ (e.g., “My feelings are sometimes so intense that I get scared.”). It is an ability subsumed under the regulation of the self and describes the capacity to tolerate unpleasant emotional states. The scale showed excellent reliability (Cronbach's α = 0.842) in our study.
- *Affect Regulation* is one of four subscales of the MZQ that consists of three items (e.g., “Often I can't control my feelings”). It describes the inability to modulate one's own affective states (Hausberg et al., 2012). The scale showed adequate reliability (Cronbach's α = 0.641) in our study.
- *Emotional communication* is a six-item subscale of the OPD-SQ (e.g., “I've been told before that I don't show my feelings enough”). It is the ability to express and communicate the own emotional states. The scale showed good reliability (Cronbach's α = 0.758) in our study.

#### Measurements of Psychological Distress and Attachment Style

To capture psychological distress, levels of depressive symptoms and anxiety symptoms were assessed via questionnaire: Depressive symptoms were measured using the nine-item depression module of the patient health questionnaire (PHQ-9, range 0–27) (Kroenke et al., 2001). It showed excellent reliability in our study (Cronbach's α = 0.871). Anxiety symptoms were assessed using the Generalized Anxiety Disorder seven-item questionnaire (GAD-7, range 0–21) (Spitzer et al., 2006). The scale showed excellent reliability (Cronbach's α = 0.905) in our study. Both questionnaires are especially recommended for phenotyping patients with IBS in large scale studies (Boeckxstaens et al., 2016).

Attachment was measured with the 12-item short version of the Experiences in Close Relationships Scale (Brennan et al., 1998) (ECR-RD12 (Ehrenthal et al., 2009), range 12–48). It captures attachment related anxiety and attachment related avoidance as two subscales of insecure attachment styles (mean values range 1–7). Both subscales showed good reliability (Cronbach's α = 0.820 and α = 0.713) in our study.

### Statistical Analyses

All analyses were carried out using IBM SPSS statistics for Windows, version 25. Patients with IBS and HCs were frequency-matched for sex and education (International Standard Classification of Education ≥2 or <2) and selected via a random sampling procedure (Wacholder et al., 1992). Missing data was <5% and randomly distributed. Missing values were replaced using mean value imputation if their frequency was below 20% (Kroenke et al., 2010). In about 2% of observations, the value was replaced by the mean value of the scale within a person (Ipsative Mean Imputation). Multivariate analysis of variances (MANOVA) was conducted using the groups (IBS vs. HCs) as independent variables and the dimensions of affective processing as dependent variables. A *post-hoc* power analysis revealed that the chosen sample size (127/group) was sufficient to detect an effect size of *f*^2^ = 0.055 (=small-medium) with 80% power and 5% alpha error. Effect sizes (d) were reported and interpreted according to Cohen (Cohen, 1988): *d* = 0.2 (small effect), *d* = 0.5 (moderate effect), *d* = 0.8 (large effect). Violin plots were visualized with the statistical programming language R with the package ggplot2. For further exploratory analyses, correlational analyses were used and scatterplots visualized the connection between IBS symptom severity and the deficits on the dimensions of affective processing. Additionally, linear regression analyses were conducted to exploratively investigate the impact of IBS symptom severity, depression, anxiety, and anxious and avoidant attachment on affective processing dimensions.

## Results

A study flow chart of the recruitment of patients with IBS and HCs has previously been published (Berens et al., 2020). Finally, *n* = 127 patients with IBS (according to ROME-III and physician) were analyzed after *n* = 1110 patients were screened for eligibility. Healthy participants were recruited online and out of *n* = 239 eligible participants *n* = 127 HCs were frequency matched to the patients with IBS.

### Patient Characteristics

Sociodemographic characteristics are presented in Table 1.

**Table 1:** Sociodemographic characteristics.

	**IBS** ***n*** **=** **127**		**HCs** ***n*** **=** **127**		*p*-value^*^
**Sociodemographic characteristics**	**Effective n**		**Effective n**		
Gender—female^a^	127	80 (63.0)	127	80 (63.0)	-
Age—years^b^	127	36.5 (13.4)	127	35.1 (13.6)	0.52
Educational level—ISCED ≤ secondary^a^	127	37 (29.1)	127	37 (29.1)	-
Nationality—German^a^	103	95 (92.2)	127	117 (92.1)	>0.99
Marital status—living with a partner^a^	126	79 (62.7)	127	66 (52.0)	0.21
Professional life, paid employment^a^	118	105 (89.0)	124	107 (86.3)	0.61

*IBS, irritable bowel syndrome; HC, healthy controls; ISCED, International Standard Classification of Education ( ≤ 2 indicates <10 years of education)*.

* *P-value calculated by chi-squared method for frequencies and Kruskal-Wallis-Test for continuous variables*.

a *absolute numbers and percentages N (%) were reported*.

b *mean values and standard deviations M (SD) were reported*.

#### Clinical Characteristics of Patients With IBS

Patients with IBS were recruited from general practitioners (11.0%), gastroenterological specialty practices (45.7%) and tertiary care outpatient clinics including IBS specialty clinic (43.3%). Mean symptom duration was 5.5 years. The subtypes were distributed as follows: IBS-C (*n* = 11, 8.7%), IBS-D (*n* = 55, 43.3%), IBS-M (*n* = 52, 40.9%), IBS-U (*n* = 9, 7.1%). IBS symptom severity was 282.8 (93.7) on average, with 15.3% having mild (IBS-SSS <175), 39.6% having moderate (IBS-SSS 175-300) and 45.0% having severe (IBS-SSS > 300) IBS symptoms. Overall, there were small correlations between IBS symptom severity and dimensions of affective processing (see scatterplots in Supplementary Figure 1). In patients with IBS, there was only a small correlation between IBS symptom severity and affect tolerance (*r* = 0.191, *p* = 0.045). In patients with IBS, levels of depressive symptoms were 8.8 (5.7), levels of anxiety symptoms were 7.1 (5.2) on average. The mean values of anxious and avoidant attachment in patients with IBS were 2.5 (1.4) and 2.2 (1.0), respectively.

#### Differences in Dimensions of Affective Processing

**Figure 1:**
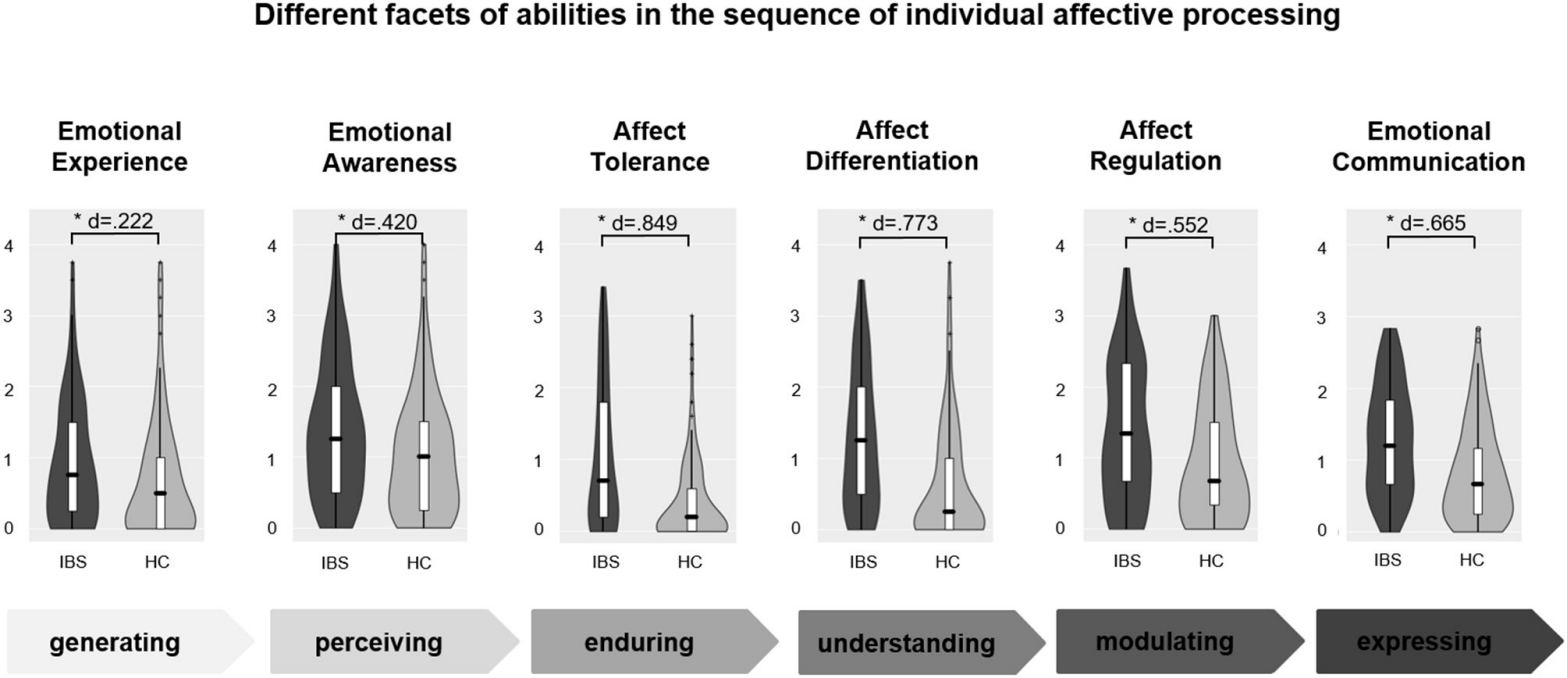
Differences in dimensions of affective processing in IBS. The figure illustrates the whole spectrum of affective processing from generating, perceiving, tolerating, understanding, modulating, and expressing of affects. All items had an answering format of 0–4 (mean value of subscales on y-axis). Higher values reflect higher deficits in affective processing. The violin plots illustrate the distribution of the data by plotting the density (violin shaped mantle) including box plots with quartiles (white box), mean values (black line), and outliers (individual data points), separated for patients with irritable bowel syndrome (IBS) and healthy controls (HC). All group differences were significant (*), Cohens d effect sizes show the amount of the effect.

Patients with IBS differed from HCs in affective processing [Pillai-Spur: *V* = 0.191, *F*(6, 246) = 9.671, *p* < 0.001]. Figure 1 shows the violin plots of the dependent variables separately for patients with IBS and HCs (see Supplementary Table 1). Patients with IBS showed higher deficits in all dimensions of affective processing, but effect sizes were largest for affect tolerance (*d* = 0.849, *p* < 0.001) and lowest for emotional experience (*d* = 0.222, *p* = 0.010) and emotional awareness (*d* = 0.420, *p* < 0.001). Moderate effect sizes were found for affect differentiation (*d* = 0.773, *p* < 0.001), emotional communication (*d* = 0.665, *p* < 0.001) and affect regulation (*d* = 0.552, *p* < 0.001). To estimate the intercorrelations between the six dimensions, the correlation matrix can be found in Supplementary Table 2.

#### Factors Influencing Deficits in Affective Processing in Patients With IBS

Six linear regression models were conducted to analyze the impact of IBS symptom severity, psychological distress (anxiety and depression) and attachment styles (anxious and avoidant) on the different dimensions of affective processing in IBS (Supplementary Table 3). All the models were significant and the factors explained between 25% (emotional awareness) and 50% of variance (affect tolerance). Anxious attachment was associated with higher deficits in affect tolerance [*t*(99) = 3.301, *p* < 0.001], affect regulation [*t*(99) = 2.789, *p* = 0.006] and emotional communication [*t*(99) = 2.197, *p* = 0.030], while avoidant attachment was associated with higher deficits in emotional experiencing [*t*(99) = 3.707, *p* < 0.001] and emotional awareness [*t*(99) = 3.223, *p* = 0.002]. The anxiety levels influenced deficits in emotional awareness [*t*(99) = 2.255, *p* = 0.026], affect tolerance [*t*(99) = 4.249, *p* < 0.001], affect differentiation [*t*(99) = 4.339, *p* = < 0.001], affect regulation [*t*(99) = 3.591, *p* = 0.001] and emotional communication [*t*(99) = 2.436, *p* = 0.017], but not emotional experiencing. The depression levels had no effect on any dimension of affective processing. IBS symptom severity was only associated with emotional communication in the way that higher symptom severity was associated with less deficits in emotional communication [*t*(99) = −2.025, *p* = 0.046].

## Discussion

The primary aim of this study was to systematically analyze different dimensions of affective processing in patients with IBS and to explore the extent of potential deficits. Therefore, we compared the whole spectrum of affective processing from generating, perceiving, tolerating, understanding, modulating, to expressing of affects between patients with IBS and HCs. Additionally, we analyzed the impact of IBS symptom severity, psychological distress and attachment styles on the different dimensions of affective processing in IBS.

We found that patients with IBS showed deficits in all six dimensions of affective processing. Interestingly, the effect was almost four times greater for affect tolerance (large effect size) than for experiencing affects (small effect size) and two-fold more than emotional awareness. Furthermore, affect differentiation, emotional regulation, and emotional communication were somewhat impaired (moderate effect sizes). These results indicate that there is a specific pattern of impaired affective processing in patients with IBS. They appear to have less of a problem with generating and perceiving their affects, but more in tolerating, understanding, regulating, and communicating affects.

Results indicate that there is also a great level of heterogeneity in the measures of affective processing and it is important to notice that not all patients with IBS are characterized by deficits in affective processing. Moreover, explorative analyses indicated that affective processing was significantly influenced by levels of anxiety while levels of depression or symptom severity seem to have only minor effects on the measures of affective processing. Additionally, anxious attachment was associated with higher deficits in affect tolerance, affect regulation, and emotional communication, while avoidant attachment was associated with higher deficits in emotional experiencing and emotional awareness. The results indicate that there are certain subtypes within patients with IBS who have specific deficits in affective processing.

Overall, this study is in line with previous studies that showed deficits in affective processing in patients with IBS (Portincasa et al., 2003; Jones et al., 2006; Elsenbruch et al., 2010; Fournier et al., 2018). In accordance with the present results, a current review has summarized that functional gastrointestinal disorders are associated with alexithymic characteristics (Kano et al., 2018). This finding broadly supports the work of other studies in this area linking somatoform disorders with impaired affect regulation (Waller and Scheidt, 2006). Contrary to expectations, however, deficits in affective processing were not most prominent in the area of emotional awareness and experiencing, but within the dimensions of affect tolerance, affect differentiation, emotional regulation, and emotional communication. This supports the concept of distinct dimensions of affective processing in IBS and raises the question of specified theoretical considerations.

### Different Facets of Affective Processing in Theoretical Models of IBS

To capture the specific facets of affective deficits, this study is based on the sequential affect cascade model of structural abilities (Rudolf, 2013). These are different structural abilities that could be disturbed separately, but also build upon or influence each other. For example, emotional awareness could serve as an important precondition to successful emotion regulation (Füstös et al., 2013). The results regarding the different affect dimensions were classified into previous literature and etiological considerations:

***Lower order generation and perception (emotional experiencing and awareness):*** According to our results, patients with IBS show only small differences in emotional experiencing and awareness compared to HCs. Classic concepts, however, emphasize the absence of emotional experience (Lipowski, 1987) and the lack of interoceptive awareness in somatoform patients (Pollatos et al., 2011) and focus on “emotional blindness” or “emotional numbness” as key mechanisms in a subgroup of patients with IBS (Salmon et al., 2003). Additional analyses of our study suggest that deficits in emotional experiencing and emotional awareness seem to be relevant in patients with IBS with high levels of avoidant attachment. Therefore, these deficits may be especially relevant for a subgroup of patients with IBS with deactivation regulation strategies resulting in an inhibition and suppression of emotions as well as an avoidance of closeness, intimacy and dependence in close relationships (Mikulincer et al., 2003). However, this concept does not go far enough to capture the key deficits in affective processing for patients with IBS in general.

***Enduring emotions (affect tolerance):***In our study, the most pronounced differences between patients with IBS and HCs were found in the dimension of affect tolerance. In line with a developmental theory of mentalizing abilities (Fonagy et al., 2018), the abilities of tolerating emotions are influenced by early interactions between the child and a caregiver. Within this context, the capacity of the caregiver to adequately capture, process, and mirror the child's affective states strongly influences the capacity of the child to form representations of former undifferentiated affective states. Deficits in affect tolerance contain the risk of emotional overload, which exceeds the individual tolerance level and can therefore no longer be adequately handled by the patient. Our additional analyses showed that deficits in affect tolerance seem to be especially relevant for patients with high rates of general anxiety and anxious attachment. Therefore, this deficit seems to be relevant for a subgroup of patients with IBS with high emotional arousal, hypervigilance for threats, and hyperactivating attachment strategies that result in overdependence on external reassurance (Mikulincer et al., 2003). Furthermore, illness anxiety can be seen as an inability to tolerate distressing emotions in an adaptive way (O'Bryan et al., 2018). In line with that, a previous study showed (illness) anxiety to be a disease-specific factor in patients with IBS (Berens et al., 2019). Further studies are needed to show a specific link between difficulties in affect tolerance, higher illness anxiety, and anxious attachment in patients with IBS.

***Higher order understanding and labeling of emotions (affect differentiation):***In this study, affect differentiation was the second largest deficit of affective processing reported by patients with IBS. In line with that a previous study showed that patients with a higher attention on somatic symptoms without the capacity of processing them showed increased symptoms (Ballespí et al., 2019). This emphasizes the importance of higher order understanding of emotions in protecting from somatic complaints. The labeling of emotions also had a calming effect, because labeling affects diminishes emotional reactivity by decreasing amygdala response and increasing activity in right ventrolateral prefrontal cortex (Lieberman et al., 2007). Therefore, our results regarding affect differentiation support previous concepts that emphasize the importance of higher cognitive-developmental processes of understanding emotions [cognitive-developmental model of emotional awareness; (Lane et al., 1990)], symbolizing emotion schemas (Bucci, 1997) or mentalizing bodily states (Fonagy et al., 2018).

***Modulation of emotions (affect regulation):***The ability to regulate affects seems to be moderately impaired according to the results of this study. Regarding our further explorations, affect regulation in patients with IBS seem to be influenced by general anxiety and attachment related anxiety. This means that at least a subgroup of patients with IBS experiences to have little control over their emotions and to have difficulties in self-calming. In keeping with this, previous studies showed top-down processes of catastrophizing to be especially relevant in patients with IBS (Lackner et al., 2004). Additionally, dysfunctional emotion regulation and coping strategies, like higher suppression and passive avoidance coping have previously been observed in patients with IBS (Jones et al., 2006). Anxiety and depression seem to be relevant mediators in the relationship between emotion regulation and somatization (Schwarz et al., 2017) that could be a relevant mechanism in IBS as well.

***Expression of emotions (emotional communication):***In this study, patients with IBS reported that their emotional communication is reduced. Emotional communication is an ability that is formed during the early dialogues with the caregiver about mental and bodily states (Harris, 1999; Rudolf and Henningsen, 2003). Classic concepts postulate that patients with functional somatic diseases communicate somatically instead of emotional. Reasons for this might be that patients with IBS held significantly more beliefs about the unacceptability of emotions compared to HCs (Bowers and Wroe, 2016). Additionally, patients with IBS tried to avoid the expression of unpleasant emotions for reasons of social desirability (Sibelli et al., 2017). It is known from studies with somatoform patients that they often have grown up in an atmosphere of emotional avoidance (Lind et al., 2014). Therefore, they are supposed to suppress and avoid emotions because of negative beliefs regarding emotions or learned reinforcements. A recent metasynthesis also emphasized interpersonal reasons for emotional avoidance like pleasing and controlling unreliable others (Krivzov et al., 2020). However, there are also indications that patients with IBS do express emotions, but more specific impairments appear. According to a previous study, patients with IBS expressed emotions like anger and sadness, but emotional expression was not consistent with the neurophysiological responses and some emotions that would have been expected (e.g., anxiety) were not reported (Fournier et al., 2018). Therefore, further studies may investigate the different dimensions of affective processing on specific emotions and integrate objective measures to subjective evaluations.

Overall, the study results reveal some specific features in the affective processing of patients with IBS compared with matched HCs. This offers interesting approaches for the development of new pathophysiological explanatory models as well as possible psychological interventions. This study had a psychosocial perspective on IBS that is in line with functional somatoform disorders and developmental theories of attachment and mentalizing processes (Waller and Scheidt, 2006; Luyten et al., 2012; Fonagy et al., 2018). Nevertheless, it must be emphasized that pathogenesis of IBS is a multifactorial process based on complex bio-psycho-social interactions (Oudenhove et al., 2016). Especially recent studies show more and more the importance of genetic, immunological as well as microbiological influences in IBS (Niesler et al., 2021). Relevant morphologic and physiological abnormalities in IBS patients include alterations in gastric motility and colonic mucosal permeability, processes of visceral hypersensitivity, low grade inflammation, altered gut microbiota composition, and altered central nervous system as well as peripheral pain processing (Elsenbruch, 2011; Drossman, 2016; Oudenhove et al., 2016). Our results do not allow any conclusions about causal relationships and must therefore not be misunderstood to interpret IBS as a purely somatoform or psychogenic clinical picture, but must be considered as one factor among many that shape the clinical picture of IBS.

### Implications for Future Studies

This study captures different dimensions of subjective problems in affective processing in line with the affect cascade model. However, there is evidence that patients with difficulties in affective processing sometimes are not aware of them (Waller and Scheidt, 2006). Further studies, therefore, should build upon these results by additionally assessing the affective abilities in a performance test [e.g., Levels of Emotional Awareness Scale (Lane et al., 1990)] or including external evaluation [e.g., Affect Consciousness Interview (Monsen et al., 1996)]. The mentalizing concept usually integrates the understanding of own and other emotions (Fonagy et al., 2018). However, this study characterizes the own affective process very well, but provides no data about understanding the emotions of others. Further studies could complement this by integrating experimental task from the context of Theory of Mind [e.g., Frith-Happé-Animation task (Abell et al., 2000) or reading the mind in the eye task (Baron-Cohen et al., 2001)]. Also, with this study we can't conclude if the difficulties in affective processing are a cause or a consequence of IBS due to the cross-sectional design. Classic approaches postulate deficits of embodied mentalization as a result of early experiences that increase the risk of functional somatic symptoms (Luyten et al., 2012). However, deficits in affective processing might also be a consequence of having chronic bodily complaints, so that the patients are less resistance to difficult affects due to permanent stress by somatic symptoms (Luyten et al., 2012; Berens et al., 2019). Future studies using longitudinal designs could investigate, if specific deficits in affective processing vary over time and e.g., interact with symptom severity, psychological distress (especially anxiety) and attachment patterns. It would be interesting to further investigate whether subgroups in the collective of patients with IBS that are characterized by specific dysfunctional patterns in affective processing can be traced back to specific etiological mechanisms or react differently to specific treatment approaches.

### Clinical Implications

Psychological treatments for patients with functional somatic complaints recommend addressing deficits in perception and interoceptive differentiation (Henningsen et al., 2018) and integrating emotional processing changes (Lumley and Schubiner, 2019). In accordance, current psychotherapeutic approaches for patients with IBS emphasize aspects of emotional awareness (Farnam et al., 2014) and emotional expression (Portincasa et al., 2003). However, our data suggest that it may be promising to go one step further. Patients with IBS experience emotions, but report difficulties in tolerating, differentiating, regulating, and communicating them. Therefore, it seems important to consider many different dimensions of affective processing when exploring or treating patients with IBS. While some approaches focus on strengthening the ability of tolerating, representing, and communicating affects (Luyten et al., 2012; Sattel et al., 2012), others make use of emotion in a more experience-based and interpretative way (Abbass et al., 2009). There is modest support that labeling affective cues reduces symptom reports in patients with IBS (Constantinou et al., 2014). Additionally, interventions that address the acceptance and expression of unpleasant emotions are supposed to be promising in patients with IBS (Bowers and Wroe, 2016). Therefore, the therapeutic focusing on affect differentiation and emotional expression seems important for this patient group as well. This again is in line with current approaches of using specific diagnoses of personality functioning to inform treatment planning and evaluation (e.g., Ehrenthal and Benecke, 2019). Furthermore, our results indicate that especially a subgroup of patients with IBS that is characterized by high anxiety and insecure attachment patterns show deficits in affective processing. This could help to identify the relevant patients that potentially profit from psychotherapeutic treatment with a focus on affective processing deficits. Taken together, we tentatively recommend incorporating these variables into current treatment approaches for IBS and other areas of somatic symptom disorders.

### Strengths and Limitations

This is a large multicenter study comparing patients with IBS and HCs in a controlled design. A representative IBS patient cohort with a physician validated diagnosis is provided. It is a main strength of this study that different dimensions of affective processing were assessed beyond the traditional concept of alexithymia. Limitations of the study are that there was not one questionnaire assessing the affective processing, but different subscales. Nevertheless, the subscales cover the relevant scope of affective processing. Furthermore, this study does not elucidate which emotions are difficult to regulate. This should be further addressed in prospective studies as well as individualized treatment approaches. Specific differences are presented here at group level which does not allow conclusions to be drawn about deficits at individual level. Patients with IBS were barely of IBS-C subtype, but mainly of IBS-D and IBS-M subtype. Additionally, we focused on psychological mechanisms of affective processing in IBS, but lack potential biological mechanisms. Finally, it is also important to emphasize that we did not look at clinical outcomes. Thus, a greater effect size does not necessarily mean a greater clinical relevance: even small deficits can have a high clinical relevance, and large deficits can have only minor clinical consequences.

### Conclusion

Overall, patients with IBS seem to primarily show deficits in the capacity of tolerating affective states and in differentiating affective states. They showed more deficits in higher order understanding and modulating affects than in lower order generating and perceiving affects. Despite classic stereotypes of low emotional experiencing and awareness, emotional hyperactivation seem to be more frequent than emotional deactivation. This is reflected in the larger deficits in affect tolerance and emotion regulation compared to the lower deficits in emotional experiencing and awareness. These results indicate to expand existing psychotherapeutic interventions for patients with IBS on the whole process of affective processing. It seems important not to limit the focus on emotional experiencing and awareness, but to especially consider deficits of affect tolerance, differentiation, regulation, and communication.

## Data Availability Statement

The raw data supporting the conclusions of this article will be made available by the authors, without undue reservation.

## Ethics Statement

The studies involving human participants were reviewed and approved by Ethics Research Committee of the Faculty of Medicine, University of Heidelberg (S-635/2016). The patients/participants provided their written informed consent to participate in this study.

## Author Contributions

SB, RS, JE, DB, WE, and JT conceived and designed the study. SB and RS obtained funding. SB and JT collected the data. SB statistically analyzed and all authors interpreted the data. SB drafted the manuscript. All authors critically revised the manuscript and provided important intellectual content. All authors approved the final version of the article.

## Conflict of Interest

JE is one of the original developers of the OPD-SQ. The remaining authors declare that the research was conducted in the absence of any commercial or financial relationships that could be construed as a potential conflict of interest.
