# Supplementary figures and images for: Different Dimensions of Affective Processing in Patients With Irritable Bowel Syndrome: A Multi-Center Cross-Sectional Study
Source: Front Psychol. 2021 Mar 29;12:625381. doi: 10.3389/fpsyg.2021.625381 (PMC8039143; doi:10.3389/fpsyg.2021.625381)

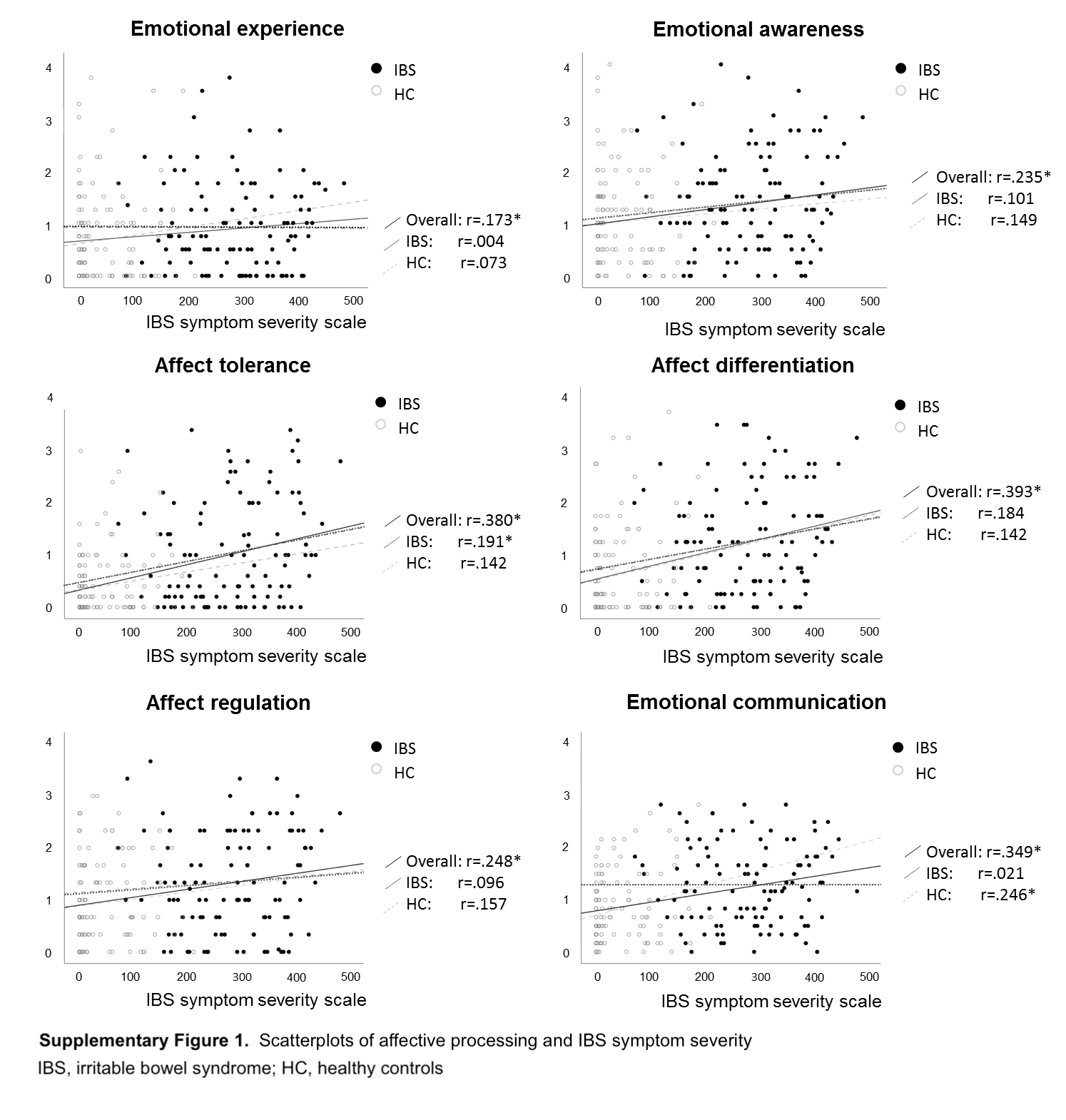

Supplement: Supplementary file 1 [file Image_1.tiff]
