# Supplementary material for: Different Dimensions of Affective Processing in Patients With Irritable Bowel Syndrome: A Multi-Center Cross-Sectional Study
Source: Front Psychol. 2021 Mar 29;12:625381. doi: 10.3389/fpsyg.2021.625381 (PMC8039143; doi:10.3389/fpsyg.2021.625381)
Supplement: Supplementary file 2 [file Table_1.docx]

**Supplement Table 1.** Differences in dimensions of affective processing

|  | **IBS**  **n=127** | | **HCs**  **n=127** | | ***p* value MANOVA** | ***Cohens d*** |
| --- | --- | --- | --- | --- | --- | --- |
| **Dimensions of affective processing** | Effective n | Mean (SD) | Effective n | Mean (SD) |  |  |
| Emotional experience | 127 | 0.94 (0.88) | 127 | 0.67 (0.84) | .01 | .222 |
| Emotional awareness | 126 | 1.43 (0.96) | 127 | 1.02 (0.87) | <.001 | .420 |
| Affect tolerance | 127 | 1.05 (0.98) | 127 | 0.40 (0.58) | <.001 | .849 |
| Affect differentiation | 127 | 1.27 (0.99) | 127 | 0.61 (0.81) | <.001 | .773 |
| Affect regulation | 126 | 1.36 (0.96) | 127 | 0.93 (0.81) | <.001 | .552 |
| Emotional communication | 127 | 1.29 (0.78) | 127 | 0.81 (0.67) | <.001 | .665 |

Abbreviations: IBS: irritable bowel syndrome; HC: healthy controls

Mean values and standard deviations M (SD) were reported.

P-values for the six different dimensions of affective processing were calculated by multivariate analysis of variance (MANOVA: Pillai-Spur: V= .191, F(6, 246)=9.671, p<.001).

Effect sizes (d) were reported: d=0.2 (small effect), d=0.5 (moderate effect), d =0.8 (large effect).
