# Supplementary material for: Different Dimensions of Affective Processing in Patients With Irritable Bowel Syndrome: A Multi-Center Cross-Sectional Study
Source: Front Psychol. 2021 Mar 29;12:625381. doi: 10.3389/fpsyg.2021.625381 (PMC8039143; doi:10.3389/fpsyg.2021.625381)
Supplement: Supplementary file 3 [file Table_2.docx]

**Supplement Table 2.** Correlation matrix between the dimensions of affective processing in IBS patients (n=127)

| **Dimensions of affective processing** | Emotional experience | Emotional awareness | Affect tolerance | Affect differen-tiation | Affect regulation | Emotional communi-cation |
| --- | --- | --- | --- | --- | --- | --- |
| Emotional experience | - |  |  |  |  |  |
| Emotional awareness | .776** | - |  |  |  |  |
| Affect tolerance | .499** | .557** | - |  |  |  |
| Affect differentiation | .697** | .744** | .801** | - |  |  |
| Affect regulation | .497** | .571** | .729** | .673** | - |  |
| Emotional communication | .632** | .533** | .564** | .609** | .467** | - |

Abbreviations: IBS: irritable bowel syndrome

Correlation coefficients r were reported; ** = p < .01
