# Supplementary material for: Different Dimensions of Affective Processing in Patients With Irritable Bowel Syndrome: A Multi-Center Cross-Sectional Study
Source: Front Psychol. 2021 Mar 29;12:625381. doi: 10.3389/fpsyg.2021.625381 (PMC8039143; doi:10.3389/fpsyg.2021.625381)
Supplement: Supplementary file 4 [file Table_3.DOCX]

**Supplement Table 3.** Linear regression models on dimensions of affective processing in patients with IBS

|  | Emotional experience | | | Emotional  awareness | | | | Affect  tolerance | | | | Affect differentiation | | | | Affect  regulation | | | | Emotional communication | | |
| --- | --- | --- | --- | --- | --- | --- | --- | --- | --- | --- | --- | --- | --- | --- | --- | --- | --- | --- | --- | --- | --- | --- |
|  | ß | t | p | | ß | t | p | | ß | t | p | | ß | t | p | | ß | t | p | ß | t | p |
| Variables |  |  |  | |  |  |  | |  |  |  | |  |  |  | |  |  |  |  |  |  |
| Constant |  | 0.703 | .484 | |  | 0.456 | .649 | |  | -0.299 | .765 | |  | 0.307 | .760 | |  | 1.489 | .140 |  | 3.115 | .002 |
| IBS symptom severity (IBS-SSS) | -.175 | -1.795 | .076 | | <.001 | 0.005 | .996 | | -.067 | -0.820 | .414 | | -.058 | -0.679 | .498 | | -.125 | -1.350 | .180 | -.195 | -2.025 | .**046*** |
| Depression (PHQ-9) | .250 | 1.617 | .109 | | -.050 | -0.314 | .754 | | .068 | 0.524 | .601 | | .030 | 0.223 | .824 | | -.051 | -0.350 | .727 | .137 | 0.903 | .369 |
| Anxiety (GAD-7) | .200 | 1.352 | .179 | | .342 | 2.255 | .**026*** | | .527 | 4.249 | **<.001*** | | .564 | 4.339 | **<.001*** | | .506 | 3.591 | **.001*** | .356 | 2.436 | **.017*** |
| Anxious attachment  (ECR-RD12) | .003 | 0.034 | .937 | | .155 | 1.568 | .120 | | .266 | 3.301 | **.001*** | | .154 | 1.823 | .071 | | .256 | 2.789 | **.006*** | .209 | 2.197 | **.030*** |
| Avoidant attachment  (ECR-RD12) | .325 | 3.707 | **<.001*** | | .289 | 3.223 | **.002*** | | .027 | 0.375 | .708 | | .129 | 1.674 | .097 | | .101 | 1.209 | .230 | .073 | 0.849 | .398 |
| **Model parameter** |  |  |  | |  |  |  | |  |  |  | |  |  |  | |  |  |  |  |  |  |
| F (5, 99) |  | 9.197 |  | |  | 7.929 |  | |  | 21.683 |  | |  | 17.867 |  | |  | 12.265 |  |  | 10.036 |  |
| p |  | **<.001*** |  | |  | **<.001*** |  | |  | **<.001*** |  | |  | **<.001*** |  | |  | **<.001*** |  |  | **<.001*** |  |
| Adjusted R^2^ |  | .283 |  | |  | . 250 |  | |  | .499 |  | |  | . 474 |  | |  | . 351 |  |  | .303 |  |

Abbreviations: IBS: irritable bowel syndrome; IBS-SSS, irritable bowel severity scoring system; PHQ-9, nine-item depression module of the patient health questionnaire; GAD-7, Generalized Anxiety Disorder seven-item questionnaire; ECR-RD12, 12-item short version of the Experiences in Close Relationships Scale.
